# Editorial: Enhancing geriatric care: international collaboration and best practices for aging populations

**DOI:** 10.3389/fmed.2025.1685777

**Published:** 2025-09-29

**Authors:** Marios Kyriazis, Peter Crome, Esther-Lee Marcus

**Affiliations:** ^1^National Gerontology Centre, Larnaca, Cyprus; ^2^Centre for Ageing & Population Studies, University College London, London, United Kingdom; ^3^School of Medicine, Keele University, Newcastle-under-Lyme, United Kingdom; ^4^Herzog Medical Center, Hebrew University of Jerusalem, Jerusalem, Israel; ^5^Faculty of Medicine, Hebrew University of Jerusalem, Jerusalem, Israel

**Keywords:** country profiles, geriatrics, older people, healthcare, health policy

## Introduction

Aging is not considered a passive course of decline but an active, multifaceted process influenced by biology, evolution, environment, technology, policy, and culture. In this Research Topic, we present studies from diverse regions—China, Singapore, Italy, Bangladesh, the Philippines, Saudi Arabia, and others. These studies offer important insights into how we can redefine and support healthy aging and highlight common challenges and regional nuances in a wide variety of age-related health topics.

Loneliness in older people has emerged as a critical yet often overlooked determinant of cognitive decline. A study entitled “*Latent profile analysis of loneliness among elderly people in the community and its relationship with cognitive function*” (Yang L. et al.) demonstrated that older individuals experiencing high levels of emotional and social loneliness were more likely to suffer cognitive impairment. This suggests a strong psychosocial-biological link that cannot be ignored in health policy. The implications are twofold: first, social interventions such as community engagement and digital inclusion are necessary; second, cognitive assessments should incorporate loneliness screening as a preventative measure.

Intrinsic capacity—a WHO-endorsed composite measure of physical and mental reserves—has emerged as a crucial metric in healthy aging. A longitudinal study from China, “*The association between sleep duration trajectories and intrinsic capacity in middle-aged and older adults in China: a longitudinal Chinese study assessing healthy aging*” (Yang C. et al.) revealed that irregular or short sleep duration over time was significantly associated with a decline in intrinsic capacity. Notably, the interaction of poor sleep and impairments in activities of daily living (ADLs) have also predicted increased depressive symptoms, underscoring the need to view sleep, functionality, and mental health as interconnected. These findings emphasize the importance of monitoring sleep patterns and ADLs collectively within national health screening programs.

The policy brief “*Brief geriatric assessments for older adults in the community in Singapore: a policy brief* ” (Tan et al.) supports the integration of simple, multidomain screening tools into primary care to identify high-risk older adults early. The streamlined nature of these tools makes them scalable and cost-effective, particularly useful for countries facing resource constraints. This model reflects a strategic policy shift: from reactive hospital care to proactive community engagement. Other nations with similar aging demographics can adopt or adapt this framework to fit local requirements.

Frailty represents a critical tipping point in aging. The paper “*Investigation of the association between the triglyceride-glucose index and the incidence of frailty among middle-aged and older adults: evidence from the China health and retirement longitudinal study*” (Long et al.) used longitudinal data from the China Health and Retirement Longitudinal Study (CHARLS) to find that metabolic dysfunction—particularly elevated triglyceride-glucose index (TyG)—predicted a higher risk of frailty. This suggests that metabolic health in midlife is a powerful modifiable factor in later-life outcomes. Public health interventions targeting blood sugar and lipid profiles may therefore delay or prevent frailty.

The comprehensive study “*Gender disparities in healthy ageing in China: current status and future prospects*” (Deng et al.) found that older women face more severe health limitations than men, despite having a longer life expectancy. This paradox—longer life but poorer health—points to systemic biases in access to healthcare, education, and financial independence. The findings advocate for gender-sensitive health policies that target women's lifelong disadvantages. Strategies could include improving education among younger women, ensuring gender equity in pension systems, and enhancing accessibility to preventive care for older women.

The observational study “*Exploring supply and demand imbalance of community-based older adult care: an observational study in Chongqing, China*” (Zhou et al.) revealed a significant mismatch between the availability of elder care services and the actual needs of older adults. In this study, it was found that many community centers were underused due to a lack of awareness, cultural reluctance, or insufficient staffing. This disconnection calls for a reassessment of care models, emphasizing not just infrastructure but also education, communication, and cultural sensitivity.

Advance care planning (ACP) is an important part of geriatric medicine, although it is often delayed until it is too late in the disease trajectory. A systematic review titled “*Implementation and effectiveness of advance care planning in hospitalized older adults with chronic heart failure: a mixed-methods systematic review and meta-analysis*” (Chen et al.) found that ACP led to improved patient satisfaction, more consistent end-of-life care, and fewer unnecessary hospitalizations. Despite the benefits, implementation barriers persist—including time constraints, provider discomfort, and lack of training. To address these issues, the authors recommend embedding ACP conversations into routine care early on, with structured tools and multidisciplinary collaboration.

The study “*The current status of trauma care for older adults in Saudi Arabia*” (Harthi et al.) highlighted systemic gaps in trauma response for the elderly, such as inadequate geriatric triage protocols and post-discharge planning. Similarly, the Taiwanese study “*Developing a novel transitional care model for older emergency department patients and exploring the target population in Taiwan*” (Hsu et al.) aimed to improve outcomes by identifying which patients would benefit most from targeted transitional care services. Both studies reinforce the need for specialized geriatric pathways within emergency and trauma wards, particularly in rapidly modernizing societies where older adults represent a growing patient cohort.

An important work, “*Clinical characteristics and outcome of very old (*≥*90 years) critically ill patients with need for intensive care after surgical intervention*” (Lücke et al.) challenges common biases of ageism in surgery. Despite their advanced age, a significant proportion of these patients survived and returned to baseline function post-operatively, especially when preoperative cognition and functionality were preserved. This situation lends credence to the argument against the tendency of blanket exclusions based on chronological age and supports a more nuanced approach based on biological age and individual prognosis.

The paper “*Factors affecting the active aging situation in Bangladesh*” (Afrin et al.) pointed to education, physical activity, social engagement, and economic independence as key contributors to active aging. Meanwhile, “*Prospects for the diagnosis and treatment of sarcopenia in the Philippines*” (Sun et al.) flagged a severe lack of diagnostic infrastructure and clinical awareness of sarcopenia—a key geriatric syndrome—despite its high prevalence. Both studies call for an urgent investment in primary care training, public health literacy, and culturally appropriate guidelines tailored to low- and middle-income countries.

Italy's long-term care reform, as analyzed in “*Reforming Italy's long-term care system: the role of barriers to and drivers of the use of services at the local level*” (Santini et al.), offers an important perspective. Despite a high-income status and aging demographic, Italy struggles with regional disparities and bureaucratic obstacles. The study identifies fragmentation of services, workforce shortages, and low public awareness as critical barriers. Solutions include decentralization of care with strong national oversight, incentives for local innovations, and clearer pathways for accessing services.

An interesting report “*Factors influencing mutual support among older people in China: a cross-sectional study*” (Xu et al.) underscores the value of community-driven care models. Older people who participate in regular activities, express willingness to give and receive help, and avoid social isolation are far more likely to engage in mutual support networks. This finding reinforces the principle that healthy aging is not solely a biomedical issue but also a social one. Community organizations, supported by policy, can act as catalysts for sustaining these networks, which, in turn, foster resilience and independence.

A diabetes and chronic pain analysis with the title “*The impact of diabetes on chronic pain in different body regions among adults aged 50 and older: a cross-sectional analysis*” (Ding et al.) adds another dimension: chronic illness not only threatens physical health but also undermines quality of life through persistent pain, even when metabolic control appears optimal. The gender- and age-specific differences—especially the higher vulnerability in women, hypertensive individuals, and those under 65—suggest that precision public health strategies are essential. Effective aging policies must therefore integrate targeted pain prevention and management into broader chronic disease care frameworks.

At life's final stage, the quality of dying and death (QODD) study “*Gender differences in quality of dying and death among older adults: a cross-sectional study in China*” (Feng et al.) reveals a gender disparity that cannot be ignored: older women report better QODD than men. Differences are partly explained by measurable variables such as place of death, number of chronic conditions, and home environment, but more than half stem from unmeasured, possibly cultural or psychosocial factors. This points to the need for gender-sensitive end-of-life care planning, which ensures equitable dignity and comfort for both men and women.

Mental health remains a thread linking physical capacity to emotional wellbeing, as demonstrated in the “*Interaction effects of sleep duration and activities of daily living on depressive symptoms among Chinese middle-aged and older adult individuals: evidence from the CHARLS*” study (Wang et al.). Both short duration of sleep and functional impairments are independently associated with depression, but their combination exerts a synergistic negative effect. Conversely, longer hours of sleep appears to buffer against the emotional impact of functional decline.

Finally, the evaluation of long-term care insurance with the title “*Evaluation of long-term care insurance pilot city policies in China: a cross-sectional study*” (Du et al.) (LTCI) in pilot cities highlights systemic factors influencing aging outcomes. While the pilots have pioneered innovative approaches, they face persistent weaknesses: narrow financing channels, mismatched supply and demand, inconsistent care quality, and insufficient oversight. Strengthening LTCI requires structural reforms—diversified funding, standardized service quality, effective monitoring—and could be further bolstered by integrating community-based models.

## Conclusions

This body of research across various continents reveals common patterns: loneliness affects cognition, sleep, and metabolic dysfunction predict frailty, and older adults—particularly women—remain underserved. In every part of the world, the fundamental needs of older people remain the same: dignity, autonomy, connection, and access to competent care.

There is no one-size-fits-all model for healthy and successful aging, but the principles of integration, early detection, cultural sensitivity, and personalization are universally applicable. Governments must move beyond reactive policy and embrace a forward-thinking, evidence-based approach grounded in these principles. We now have the tools, data, and models to build a more inclusive and compassionate future for aging. What remains is the will to act.

